# A Focus of Rumenal Cancer in Kenyan Cattle

**DOI:** 10.1038/bjc.1971.10

**Published:** 1971-03

**Authors:** W. Plowright, C. A. Linsell, F. G. Peers

## Abstract

A minimal incidence rate of 2·5% of rumenal cancer of cattle in the Nasampolai valley of Kenya Masailand has been established.

Carcinoma of the oesophagus and oesophageal region of the stomach in two free-living giant forest hogs from the same area is reported.

The high incidence of the bovine disease is thought to be associated with the abnormal forest grazing of the cattle.

The possible aetiology of the disease is discussed.


### A FOCUS OF RUMENAL CANCER IN KENYAN CATTLE

W. PLOWRIGHT, C. A. LINSELL AND F. G. PEERS

*From the East African Veterinary Research Organization, Muguga, P.O. Kabete, Kenya; the Nairobi Regional Centre of the International Agency for Research on Cancer, P.O. Box 6831, Nairobi, Kenya; and the Tropical Products Institute, London*

Received for publication October 19, 1970

**SUMMARY.—A minimal incidence rate of 2·5% of rumenal cancer of cattle in the Nasampolai valley of Kenya Masailand has been established.**

**Carcinoma of the oesophagus and oesophageal region of the stomach in two free-living giant forest hogs from the same area is reported.**

**The high incidence of the bovine disease is thought to be associated with the abnormal forest grazing of the cattle.**

**The possible aetiology of the disease is discussed.**

In 1955, Plowright reported that the residents of a valley in the Sakutiek area of the Narok district of Kenya Masailand recognized that a number of their cattle died from a disease which they claimed to diagnose clinically 6 to 36 months before death or slaughter. The disease was sufficiently common to cause grave economic loss and the annual incidence was said to be as high as 10% of the cattle population, causing some owners with grazing lands elsewhere to move their cattle from the valley. The character of the disease was established by finding squamous cell carcinoma of the rumen and oesophagus in three cases following field autopsies. No further investigation took place until September 1968, when during a preliminary visit, four clinical cases were presented and retrospective enquiry indicated that the incidence had not greatly changed. The immediate slaughter of one of these cases and subsequent post-mortem examination again showed a carcinoma of the rumen with secondary deposits in the regional lymph nodes. Since February 1969, a Masai field worker has been employed to gather data on the cattle population and grazing areas. He has also made periodic checks on the appearance and development of new cases, collecting tissues for histological examination when it was not possible for autopsy to be carried out by one of us.

### *The Valley of Nasampolai*

This somewhat remote valley is on the south-western slopes of the Mau Escarpment at approximately 36° 07′ E., 0° 50′ S. The valley lies at altitudes between 9000–10,000 feet and runs into the forested Mau hills. The valley floor is approximately 5 square miles in area and a small stream runs through it. The sides of the valley are steep and dense bamboo forest is present only 200 feet above the valley floor in some places.

Applications for reprints should be made to: The Nairobi Regional Centre, P.O. Box 6831, Nairobi, Kenya.

*The animal population of Nasampolai*

The human population of Nasampolai in February 1969, was 384 distributed between 11 " bomas ", each consisting of the living quarters of family groups and fenced compounds for confining animals at night to prevent losses from predators.

The total number of cattle at risk in February 1969, was 961, of which 252 were classed as " calves "—*i.e.* animals up to 6–7 months old, whilst 132 were " heifers " —*i.e.* female animals not more than 3 years old which have never calved. Part of the total of 99 " bullocks " (castrated males) was also probably less than 2 years old and hence about half of the total cattle population was immature. Also occupying the same area were 1792 sheep, 304 goats and 209 donkeys. A further animal census in November 1969, did not show any significant changes in this cattle population or herd structure.

The forested areas on the ridges towards the periphery of the valley harbour a number of large wild ungulates and carnivores, including buffalo (*Syncerus caffer*, Sparrman), giant forest hog (*Hylochoerus meinertzhageni*, Thomas), bush-pig (*Potamochoerus porcus*, Linnaeus), hyena (*Hyaena hyaena*, Meyer), and leopard (*Panthera pardus*, Linnaeus).

*Animal husbandry in Nasampolai*

The domestic animals are taken from the night " bomas " to their grazing every morning and returned each evening, calves being retained near the " boma " for the first 6 months or so and then accompanying the herds to more distant grazing. In this district there is no allocation of grazing rights to individual families, as is usual elsewhere in Masailand, and all grazing is communal and essentially over-stocked. Because the population of goats and sheep is so large and they graze, of necessity, on the valley floor, pasturing of cattle in partially cleared forest is often necessary although it is recognized as an unsatisfactory source of food. Whilst grazing is communal, the owners of some " bomas ", by reason of their marginal position in the valley, are forced to use high forest clearings to a greater degree than their more fortunate neighbours lower in the valley. Supplementary feeding is not practised with the exception that maize stalk residues are fed in small quantities and that earth from a natural " lick " is periodically provided to prevent a form of marasmus, possibly due to phosphorus and/or cobalt–copper deficiency. Osteophagia by cattle is also reported by the owners.

*Clinical course of the disease*

It must be stressed that the first clinical diagnosis of all cases has been made by the Masai themselves. Cattle are their main source of wealth and, indeed, subsistence, and great interest is taken in all matters pertaining to animal husbandry. They are familiar with a wide range of animal diseases and it is only due to their continued co-operation that this study has been possible. It is their custom to examine carefully the viscera of all animals slaughtered and illness in their cattle is the subject of communal consultation.

This disease at Nasampolai is sufficiently common to have acquired a vernacular name—" Embonget "—which simply refers to the rumenal tympany described below. In every case made available to us so far, the clinical diagnosis of the Masai has been supported by autopsy and subsequent histological examination.

The clinical signs which characterize the disease are:

(i) Apparent pain and difficulty in swallowing or regurgitation of food for rumination. This may be accompanied by arching of the neck. Some animals regurgitate watery rumenal contents which dribble from the mouth and nostrils as if vomiting.

(ii) Recurrent rumenal tympany, easily visible as a distention in the left flank region.

(iii) Abdominal pain evidence by grunting, trismus, arched back and slow, stiff movements. Partial anorexia and slow eating are noted occasionally.

(iv) Loss of condition in advanced cases, with the hair-coat harsh and dry and the skin tightly adherent to underlying structures.

(v) Excessive thirst is sometimes recorded.

We were at first informed that death usually occurred 6 to 9 months after the first clinical signs, the terminal stages being marked by a progressive weakness and cachexia. Some cases were said to run a longer clinical course of 2 to 3 years with periods of remission but this is difficult to confirm. More recently, constant surveillance has shown that the overt disease in some animals develops rapidly and slaughter may become imperative in a month or even less.

*Pathological findings*

A summary of the autopsy findings is found in Table I, and a detailed description of the histopathology and cytology of the lesions will be published at a later date. Thirteen of the 20 autopsies were carried out by the field assistant who was instructed to fix a wide range of tissues whether apparently normal or not.

The mouth cavity showed no abnormalities but small papillomata occurred on the mucosae of the pharynx or soft palate in a few cases. The oesophageal mucosa

TABLE I.—*Autopsy Findings in Cattle and Giant Forest Hogs from the Nasampolai Valley*

| Case No. | Sex | Age (years) | Squamous cell ca. | | Regional node metastases | Distant metastatic site | Papillomata | |
|---|---|---|---|---|---|---|---|---|
| | | | Oesophagus | Rumen | | | Upper G.I. tract | Bladder |
| 1 | F | 4–5 | 0 | + | + | 0 | 0 | 0 |
| 2 | F | 5 | 0 | + | 0 | 0 | + | 0 |
| 3 | F | 7–8 | 0 | + | + | Liver | 0 | 0 |
| 4 | F | ? | 0 | + | 0 | 0 | 0 | 0 |
| 5 | F | 7–8 | + | + | 0 | 0 | + | 0 |
| 6 | F | ? | 0 | + | 0 | 0 | + | 0 |
| 7 | F | ? | 0 | + | 0 | 0 | 0 | 0 |
| 8 | M | ? | + | + | 0 | 0 | 0 | 0 |
| 9 | F | ? | 0 | + | 0 | 0 | + | 0 |
| 10 | F | ? | 0 | + | + | 0 | + | 0 |
| 11 | M | 5 | 0 | + | 0 | Lung, liver | 0 | + |
| 12 | F | 6 | + | + | 0 | 0 | + | + |
| 13 | F | 4–5 | 0 | + | 0 | 0 | + | 0 |
| 14 | M | 5 | 0 | + | 0 | 0 | + | 0 |
| 15 | F | 6–7 | 0 | + | 0 | 0 | 0 | 0 |
| 16 | F | 4–5 | 0 | + | 0 | 0 | 0 | 0 |
| 17 | F | 9 | 0 | + | 0 | 0 | + | 0 |
| 18 | F | ? | 0 | + | 0 | 0 | 0 | 0 |
| Giant Forest Hog—*Hylochoerus meinertzhageni*, Thomas | | | | | | | | |
| | | | | Stomach | | | | |
| 19 | F | (Aged) | + | + | 0 | Lung | + | 0 |
| 20 | M | ? | + | + | 0 | Thyroid | + | ? |

in half the bovine cases exhibited papillomata, usually pedunculated and found especially in the intrathoracic region within about 20 cm. of the cardiac opening. In three cases there were elongated or circular foci of brownish erosion which represented areas of undoubted malignant change; gross thickening of the oesophageal wall, as observed and illustrated earlier (Plowright, 1955) was not seen, however, in the present series. In several cases, brownish, roughened lesions showed hyperplastic epithelium with marked activity and downgrowth of the basal cell layers and sub-epithelial mononuclear-cell reaction. Although not frankly malignant, these changes could be interpreted as " carcinoma *in situ* " or " precancerous ".

Histological examination revealed that squamous cell carcinoma of the rumen was present in every case, an invariable site being the anterior wall of the dorsal sac of the rumen, an area which is characterized by having no distinct mucosal papillae (the atrium). Lesions were also sometimes present at the cardiac opening and on the oesophageal groove; in a few cases, the pillars of the rumen were the site of both carcinomata and papillomata. Macroscopically, the tumours were of both the ulcerative and fungating types and appeared to be multicentric in origin. Wide infiltration of the rumenal wall was common, in some cases extending through the muscle layers to the sub-serous connective tissue.

In each case, many lymph nodes on and near the rumen and oesophagus were examined for metastases, both macroscopically and histologically. Secondary carcinomata were found in only 4 animals, the glands involved being particularly those of the atrial and posterior mediastinal groups (Sisson and Grossmann, 1940). Large and numerous metastases were also found in the livers of 2 animals and the lung tissue of 1 case. Two animals showed papillomata of the bladder mucosa but these did not exceed a few millimeters in diameter and were not apparently associated with gross haematuria. In case No. 13 there were haemorrhagic, eroded areas in the bladder which reached 1 × 2 cm. in size and were associated with gross, oedematous thickening of the wall; this animal must have exhibited clinical signs of haematuria.

*The incidence of rumenal cancer in Nasampolai*

The cattle owners claim that the disease has been recognized since 1935 and that the incidence has increased over the years, particularly since 1942 (Plowright, 1955). The name of the valley, Nasampolai, in the Masai dialect, is associated with excessive salivation. This and the fact that rumenal cancer has a vernacular name, " Embonget ", indicates that this is a long-standing disease well understood by the Masai. In 1955, it was estimated that the disease affected 10% of the total cattle annually but it is well known that animal holdings are not usually fully declared. The cattle population in the same year was reported to be 250 and in September 1968, to be between 500 and 600, whereas a physical check in February 1969, revealed 961. A later animal census in November 1969, gave a total of 982 and indicated some movement of cattle to and from the valley.

In the first 15 months of the field survey, 18 cases have been proven histologically and of the additional clinical cases reported, at least 12 were traced and the diagnostic criteria checked by the field worker. Therefore, in Nasampolai, we have good evidence for a minimum annual loss due to rumenal cancer of 2·5% of the total cattle population or 5% of the adult animals.

Cattle owners in several neighbouring valleys were interrogated and although a few cases of a similar disease were reported during 1969, the animals were stated to have been pastured in Nasampolai earlier in life and developed the disease within 6 months of translocation. In the next valley, Nosupukia, the residents recognize the disease clinically and at slaughter; it was said to have occurred there first in 1955 but they have only lost 8 animals from this cause since then. Healthy animals brought into Nasampolai are said to acquire the disease within as little as a year after transfer. Cattle which develop " Embonget " subsequent to transfer from the valley do not seem to introduce the disease to cattle in an unaffected area.

There is no seasonal predominance of new cases but the Masai claim that the incidence is higher as the result of prolonged periods of drought and when forest grazing is used more frequently. An estimate of the age of affected animals was obtained in 10 cases (Table I) and whereas previously the disease was seen predominantly in females 7–8 years old, in the current series it was not uncommon in cows suckling a second calf (*i.e.* about 4–5 years old). Both male and female cattle are susceptible; the predominance of females (Table I) can probably be attributed to the four-fold greater number of female animals retained in the herds.

The Masai insist that the disease is not seen in goats and sheep, these animals grazing, as already noted, on the grasses of the valley floor but never in the forest area. The over-grazing of the valley floor by goats and sheep leaves only a tough, dense, tussock grass which is generally unpalatable to cattle.

Cases of rumenal cancer in cattle are not, to our knowledge, reported from other areas in Kenya or East Africa and, as far as can be ascertained, oesophageal and stomach cancer are not recognized in the human population of Nasampolai. Similarly, no reports have been received of a disease resembling " Embonget " in the numerous buffaloes which inhabit the higher forested areas, or in bushpigs which are fairly common.

*The occurrence of stomach cancer in Giant Forest Hogs in Nasampolai*

In September 1968, some of the Masai elders informed us that they associated the disease in cattle with the presence in the area of giant forest hogs. One man clearly remembered having seen a case in this species confirmed at post-mortem examination about 15 years previously. During the period under review, fixed tissues were obtained from 2 giant forest hogs which were speared after they had been observed to show signs of illness, including abdominal tympany in 1 animal. In each case, there were large, ulcerated areas in the mucosae of the oesophagus and in the oesophageal region of the stomach. In these areas, which attained several cm. in diameter, there was often gross thickening of the wall and keratinized papillomata.

In both oesophageal and stomach lesions some epitheliomatous cell cords were seen in direct continuity with the hyperplastic or papillomatous epithelium. Small secondary deposits were found in the lung of the first animal and malignant cell islets infiltrated the thyroid gland tissue in the second (Table I). In addition, the first case exhibited an irregular enlargement of the liver which histologically was shown to be due to haemorrhagic necrosis and regenerative changes, typical of a sub-acute toxic hepatitis.

### DISCUSSION

Few cancer surveys of large cattle populations have been related to the numbers at risk and it is not possible to establish absolute incidence rates except in the series reported by Monlux *et al.* (1956) in Colorado, Misdorp (1967) in Holland, and recently, Anderson *et al.* (1969) in Britain. The crude incidence rates from these surveys vary between 23 and 60 per 100,000 animals. It is usually considered that the economic slaughter of ageing animals changes the structure of the population so radically that the number of animals of susceptible age at risk is effectively reduced. However, of more than one thousand million adult animals slaughtered for food in the United States during the period 1955–62, 80% were slaughtered under the supervision of the Department of Agriculture and 227 cattle per 100,000 were condemned with a diagnosis of neoplasia (Brandly and Migaki, 1963). This crude incidence is much higher than in the animal series quoted earlier and, indeed, higher than that reported in man, particular if adjusted for comparable physiological age (Steele, 1963). The frequency ratios in these various series are very different, cancer of the endometrium being very common in the United States, as indeed is squamous cell carcinoma of the conjunctiva and eyelid. The importance of lymphosarcoma and its geographical pattern is well recognized.

However, so far as rumenal cancer is concerned, this appears to be extremely rare in cattle. Misdorp (1967) found one case in 208 bovine tumours from an estimated cattle population of 340,000 and Anderson *et al.* (1969) record one case in 302 tumours from a survey of 1·3 million cattle. Smith and Jones (1966), reporting on a series of 1371 bovine tumours from a number of sources, also recorded only one case and comment that such neoplasms are almost non-existent. An analysis of 1000 consecutive cattle tumours from the U.S. study of Brandly and Migaki (1963) did not reveal a single squamous cell carcinoma of the rumen or oesophagus. Moulton (1961) considers cancer of the stomach rare in all domestic animals but states that cancer of the oesophagus and, in particular, papillomata, are occasionally seen in cattle. Cancer of the oesophagus is well recognized in elderly cats, Cotchin (1962) reporting 13 cases in a series of 66 feline tumours of the gastro-intestinal tract.

The minimal incidence of rumenal cancer of cattle in Nasampolai—2500 per 100,000—is, therefore, exceptional.

The incidence of cancer in wild animals is even less well documented and we must rely on data acquired under the artificial conditions of zoos. Ratcliffe (1963) states that cancer has been encountered in many captive animals and analysing material from the Philadelphia Zoo, Snyder and Ratcliffe (1963) found 100 cancers in 1702 autopsies, 284 of which were of Bovidae. No gastro-intestinal cancer in Bovidae was seen, although 2 cases were noted in the Felidae and one each in the families Mustelidae and Viverridae. Heuschele and Herrick (1962) have reported a case of cancer of the oesophagus in an antelope (*Antilope cervicapra*) in a zoo. The present report of 2 cases of squamous cell carcinoma of the oesophagus and stomach in free-living giant forest hogs from Nasampolai in one year must, we consider, be unique. It is of interest that both ruminants and monogastric animals at risk have developed this carcinoma at a similar anatomical site within this small geographical area.

Dobereiner *et al.* (1967) reported numerous cases of carcinoma, primarily of the pharynx, in cattle in Brazil associated with papillomata of the bladder and enzootic haematuria. They associated these bladder lesions with the ingestion of bracken fern and there is experimental evidence to support this suggested aetiology as gastro-intestinal cancer was recorded in long-term feeding experiments with bracken (Evans, 1968). The incidence of enzootic haematuria is world-wide

(Pamukcu, 1963) whereas gastro-intestinal cancers have not been reported from areas where it is common and where animal inspection is of a high standard. Enzootic haematuria is not infrequent in high altitude areas of Kenya (Mugera and Nderito, 1969) and bracken fern is found in the Nasampolai valley but it is unlikely to cause " Embonget " as it does not form an item of cattle forage and clinical enzootic haematuria has not been reported by the Masai.

Oesophageal tumours in sheep have been reported from South Africa, associated with a nicotine–copper sulphate drench and grazing at an altitude of about 4000 feet (Schutte, 1968): no histological identification of the tumours was obtained, however. In Nasampolai, the Masai are adamant that neither sheep nor goats contract the disease and they connect this with the fact that they are never herded in the forest. It is impossible to obtain any idea of the incidence of cancer in the giant forest hog which confines its feeding mainly to the forest flora but our findings of two cases in such short time supports the hypothesis that it is relatively common and associated with the ingestion of the same plants that cause rumenal cancer in cattle.

The Masai identify by name about 30 broad-leaved plants and grasses which cattle eat and a botanical survey of this forest flora is in progress. As scientific identification requires specimens of the plants at all stages of growth, the survey is not yet complete and the final results will be reported elsewhere.

We tentatively put forward the hypothesis that a potent carcinogen is either ingested with the forest plants at Nasampolai or rapidly produced in the stomach from precursors. In this connection the discovery of the carcinogenic properties of dimethyl-nitrosamine in rats by Magee and Barnes (1956) has stimulated an expanding literature on a range of nitroso-compounds recently reviewed by Magee and Barnes (1967) and Druckrey *et al.* (1967). The rate of administration, dosage regime, type of nitrosamine and host animal, can all affect the site of the primary lesion. Oesophageal tumours have been produced, mainly in rats, by 13 different N-nitroso-compounds out of the 29 nitroso- and chemically related azoxy-compounds listed by Magee and Barnes (1967) and Druckrey *et al.* (1967) report oesophageal tumours in rats after treatment with more than 25 nitroso-compounds. Many of the nitroso-compounds that have evoked oesophageal tumours could be expected to produce a lesion at the site of administration because of their potentially high reactivity but Druckrey *et al.* (1963*a*) have pointed out that lesions in the mouth and pharynx are uncommon with animals on a *per os* regime of various nitrosamines. Oesophageal tumours have also been produced in rats by three different routes of administration of N-nitroso-piperidine (Druckrey *et al.*, 1967).

The possible dangers of nitrosamine compounds in the environment were first pointed out by Druckrey *et al.* (1963*b*) but apart from the potential hazard in the chemical industry and to laboratory workers, these compounds were mainly of interest as laboratory carcinogens until the report by Ender *et al.* (1964) of an outbreak of acute toxic hepatitis in sheep. This outbreak was associated with the feeding of nitrite-preserved fishmeal which was subsequently demonstrated to contain dimethyl-nitrosamine (Sakshaug *et al.*, 1965). Burrell *et al.* (1966) have correlated high nitrate accumulation in molybdenum-deficient plants with the high incidence of human oesophageal cancer in localized areas of the Transkei region of South Africa and, more recently, Du Plessis *et al.* (1969) have demonstrated the presence of dimethyl-nitrosamine in the ripe fruit of *Solanum incanum* grown on such molybdenum-deficient soils. These fruits are said to be used to sour milk and human dietary exposure to nitrosamines could, therefore, have arisen. Before this the natural occurrence of compounds of this type in plant material likely to be involved in human and animal diets was, apparently, limited to cycasin (reviewed by Whiting, 1963).

Excessive nitrate accumulation can occur in plants and this could give rise to nitrite by reduction during rumenal metabolism. Acute nitrate poisoning leading to methaemoglobinaemia in cattle has been reported and in some cases the formation of nitrosohaemoglobin has been demonstrated (Case, 1957). Sodium nitrite itself is not carcinogenic on prolonged feeding to rats (Druckrey *et al.*, 1963*b*). Recent reviews by Magee and Barnes (1967), *Lancet* (1968), and Lijinsky and Epstein (1970) have underlined the possible dangers of nitrosamine compounds in the environment. Lijinsky and Epstein (1970) have stressed that the presence of nitrosatable secondary amines is possibly more limiting than nitrate or nitrite for the *in vitro* production of nitrosamines during cooking or for their *in vivo* production under physiological conditions in the mammalian stomach. Sander (1967) has investigated the possibilities of nitrite in human nutrition leading to the formation of nitrosamines in the stomach and Sander *et al.* (1968) demonstrated the formation of nitrosamines in rat stomachs in *in vivo* experiments and also that the degree of nitrosation depends upon the basicity of the secondary amine concerned. Sander and Seif (1969) demonstrated that nitrate can be reduced to nitrite in human stomachs by bacterial action and that the conditions were then optimal for the formation of nitrosamines from secondary amines. Sen *et al.* (1969) have demonstrated the *in vitro* formation of diethyl-nitrosamine by the incubation of nitrite and diethylamine with the gastric juices from rats, rabbits, cats, dogs and man, and nitrosation was also shown to occur *in vivo* in cats and rabbits. Mirvish (1970) has studied the kinetics of dimethylamine nitrosation and estimated the likely amounts of dimethyl-nitrosamine formation in foods during storage and in the human stomach from the consumption of foods containing nitrite and dimethylamine.

Oesophageal tumours have been produced in rats by the combined feeding of nitrite and a secondary amine, methylbenzylamine (Sander, 1968) and liver tumours have been evoked by a regime of nitrite plus morpholine (Sander, 1969). This author has also induced neurogenic tumours in rats fed sodium nitrite and $NN^1$ dimethylurea (Sander, 1970).

Magee and Barnes (1967), commenting on Plowright's original observation in 1955, speculated that a nitroso-compound could be present in one of the plants consumed by the cattle. Botanically, the Valley shows no immediately obvious differences from other nearby valleys and we consider that the socio-economic situation in Nasampolai whereby the Masai use forest grazing atypical of normal cattle grazing, is the probable reason for the high incidence of this cancer within such a small community. If nitroso-compounds are involved in the aetiology of this focus of rumenal cancer in the cattle of Nasampolai, then they could either be present in the plants as eaten or be formed *in vivo* during rumenal digestion. These possibilities are being investigated.

We thank Dr. Maloiy of the East African Veterinary Research Organization for his considerable help and advice, Dr. Greenway of the East African Agriculture and Forestry Research Organization for extensive botanical investigations, and Joseph ole Swakei, our field worker, whose tact and enthusiasm have made this study possible.

The work has been financed largely from funds made available by the Nairobi Regional Centre of the International Agency for Research on Cancer.

### REFERENCES

ANDERSON, L. J., SANDISON, A. T. AND JARRETT, W. F. H.—(1969) *Vet. Rec.*, **84**, 547.

BRANDLY, P. J. AND MIGAKI, G.—(1963) *Ann. N.Y. Acad. Sci.*, **108**, 872.

BURRELL, R. J. W., ROACH, W. A. AND SHADWELL, A.—(1966) *J. natn. Cancer Inst.*, **36**, 201.

CASE, A. A.—(1957) *J. Am. vet. med. Ass.*, **130**, 323.

COTCHIN, E.—(1962) *Proc. R. Soc. Med.*, **45**, 671.

DOBEREINER, J., TOKARNIA, C. A. AND CANELLA, C. F. C.—(1967) *Pesq. Agropec. Bras.*, **2**, 489.

DRUCKREY, H., PREUSSMAN, R., BLUM, G., IVANKOVIC, S. AND AFKHAM, J.—(1963*a*) *Naturwissenschaften*, **50**, 100.

DRUCKREY, H., PREUSSMAN, R., IVANKOVIC, S. AND SCHMÄHL, D.—(1967) *Z. Krebsforsch.*, **69**, 103.

DRUCKREY, H., STEINHOFF, D., BEUTHNER, H., SCHNEIDER, H. AND KLÄRNER, P.—(1963*b*) *Arzneimittel-Forsch.*, **13**, 320.

DU PLESSIS, L. S., NUNN, J. R. AND ROACH, W. A.—(1969) *Nature, Lond.*, **222**, 1198.

ENDER, F., HAVRE, G., HELGEBOSTAD, A., KOPPANG, N., MADSEN, R. AND CEH, L.—(1964) *Naturwissenschaften*, **51**, 637.

EVANS, I. A.—(1968) *Cancer Res.*, **28**, 2252.

HEUSCHELE, W. P. AND HERRICK, W. A.—(1962) *J. Am. vet. med. Ass.*, **141**, 486.

LANCET—(1968) Leading Article, i, 1071.

LIJINSKY, W. and EPSTEIN, S. S.—(1970) *Nature, Lond.*, **225**, 21.

MAGEE, P. N. AND BARNES, J. M.—(1956) *Br. J. Cancer*, **10**, 114.—(1967) *Adv. Cancer Res.*, **10**, 163.

MIRVISH, S. S.—(1970) *J. natn. Cancer Inst.*, **44**, 633.

MISDORP, W.—(1967) *J. comp. Path. Ther.*, **77**, 211.

MONLUX, A. W., ANDERSON, W. A. AND DAVIES, C. L.—(1956) *Am. J. vet. Res.*, **17**, 646.

MOULTON, J. E.—(1961) 'Tumours in Domestic Animals'. Berkeley and Los Angeles (University of California Press).

MUGERA, G. M. AND NDERITO, P.—(1969) *J. comp. Path. Ther.*, **79**, 251.

PAMUKCU, A. M.—(1963) *Ann. N.Y. Acad. Sci.*, **108**, 938.

PLOWRIGHT, W.—(1955) *J. comp. Path. Ther.*, **65**, 108.

RATCLIFFE, H. L.—(1963) *Am. J. Cancer*, **17**, 116.

SAKSHAUG, J., SÖGNEN, E., HANSEN, M. A. AND KOPPANG, N.—(1965) *Nature, Lond.*, **206**, 1261.

SANDER, J.—(1967) *Arch. Hyg. Bakt.*, **151**, 22.—(1968) *Hoppe-Seyler's Z. physiol. Chem.*, **349**, 429.—(1969) *Z. Krebsforsch.*, **73**, 54.—(1970) *Arzneimittel-Forsch.*, **20**, 418.

SANDER, J., SCHWEINSBURG, F. AND MENZ, H. P.—(1968) *Hoppe-Seyler's Z. physiol. Chem.*, **349**, 1691.

SANDER, J. AND SEIF, F.—(1969) *Arzneimittel-Forsch.*, **19**, 1091.

SCHUTTE, K. H.—(1968) *J. natn. Cancer Inst.*, **41**, 321.

SEN, N. P., SMITH, D. C. AND SCHWINGHAMER, L.—(1969) *Fd Cosmet. Toxic.*, **7**, 301.

SISSON, S. AND GROSSMAN, J. D.—(1940) 'The Anatomy of Domestic Animals', 3rd edition. Philadelphia (W. B. Saunders & Co.).

SMITH, H. A. AND JONES, T. C.—(1966) 'Veterinary Pathology', 3rd edition. Philadelphia (Lea & Febiger).

SNYDER, R. L. AND RATCLIFFE, H. L.—(1963) *Ann. N.Y. Acad. Sci.*, **108**, 793.

STEELE, J. H.—(1963) *Ann. N.Y. Acad. Sci.*, **108**, 880.

WHITING, M.—(1963) *Econ. Bot.*, **17**, 270.


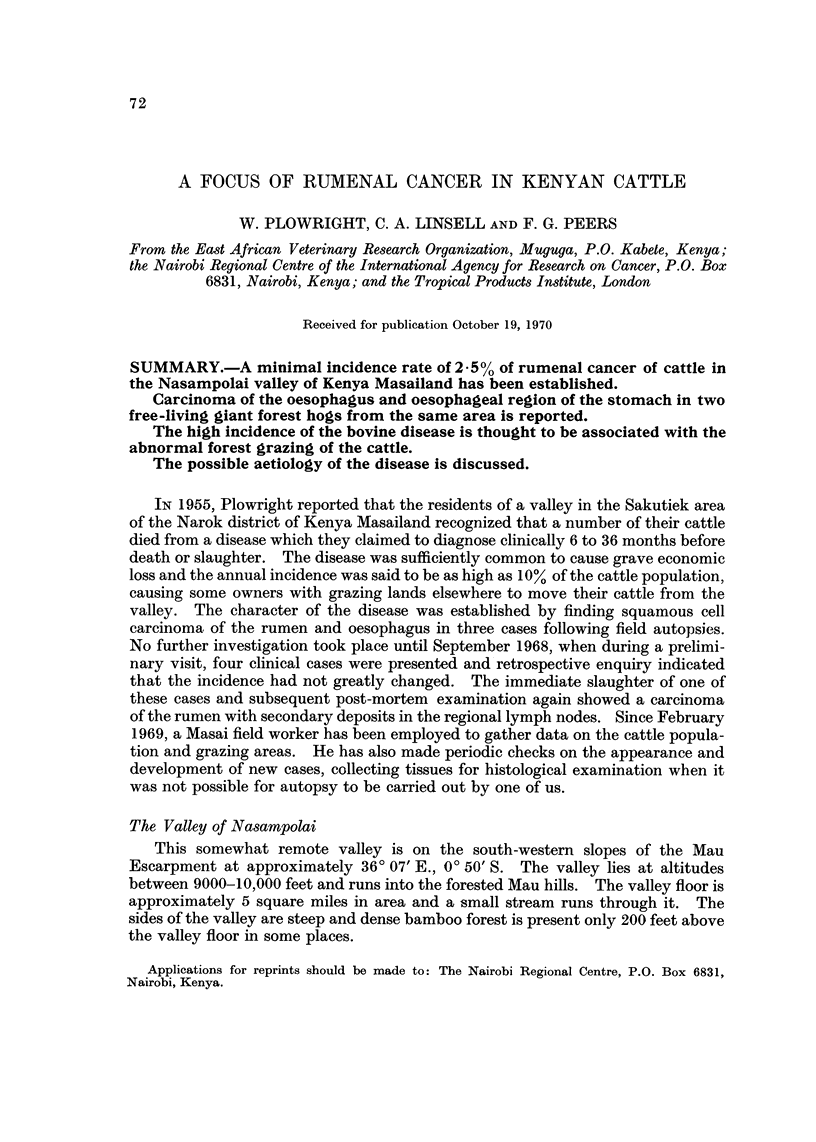

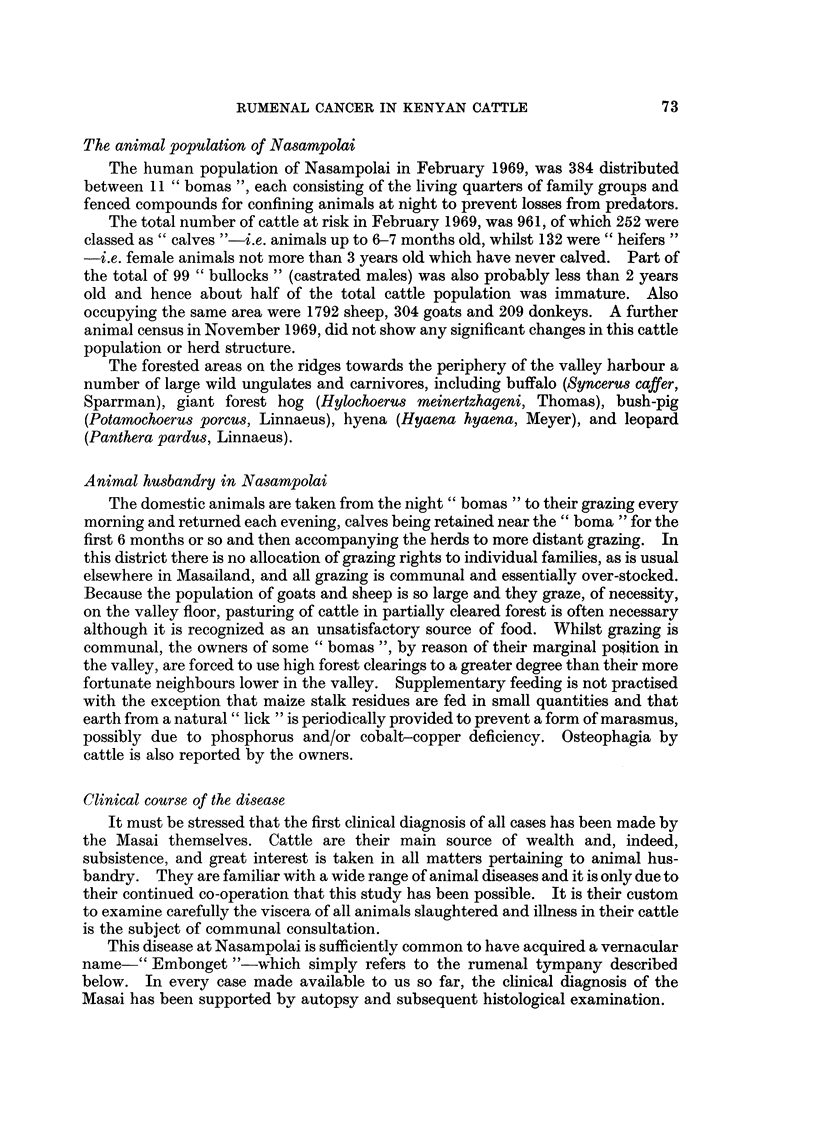

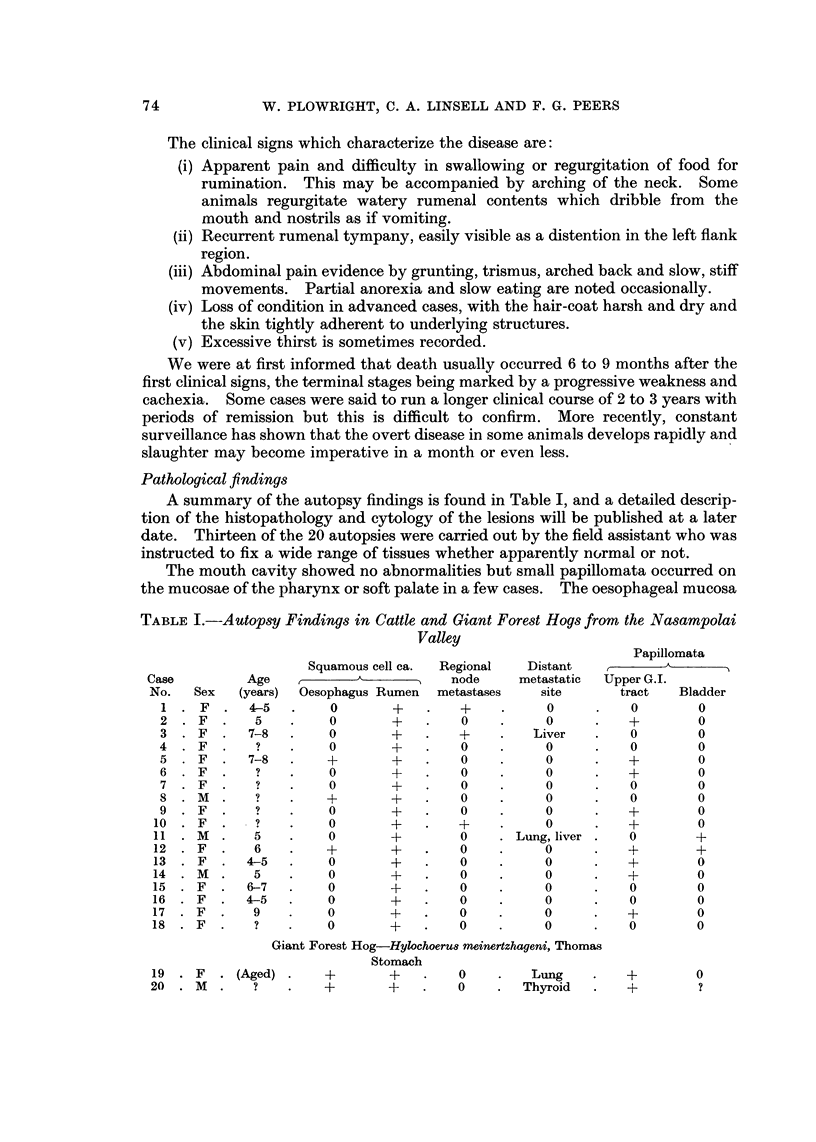

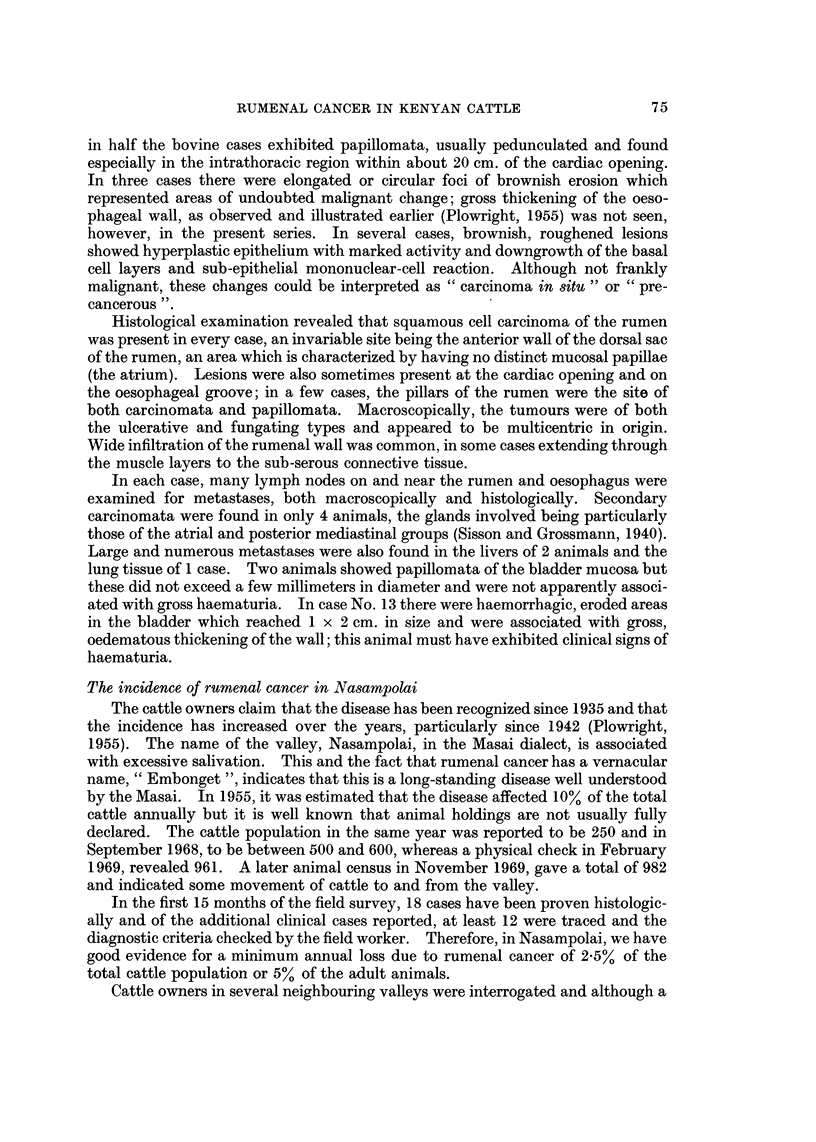

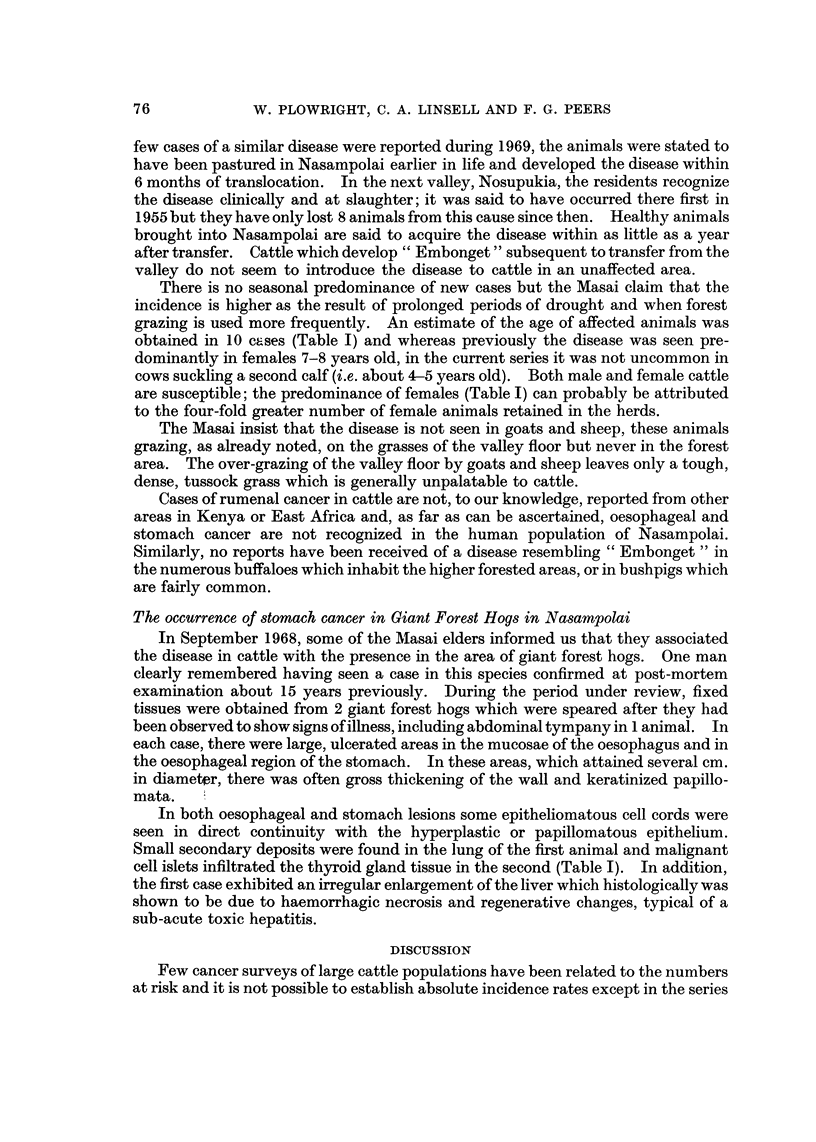

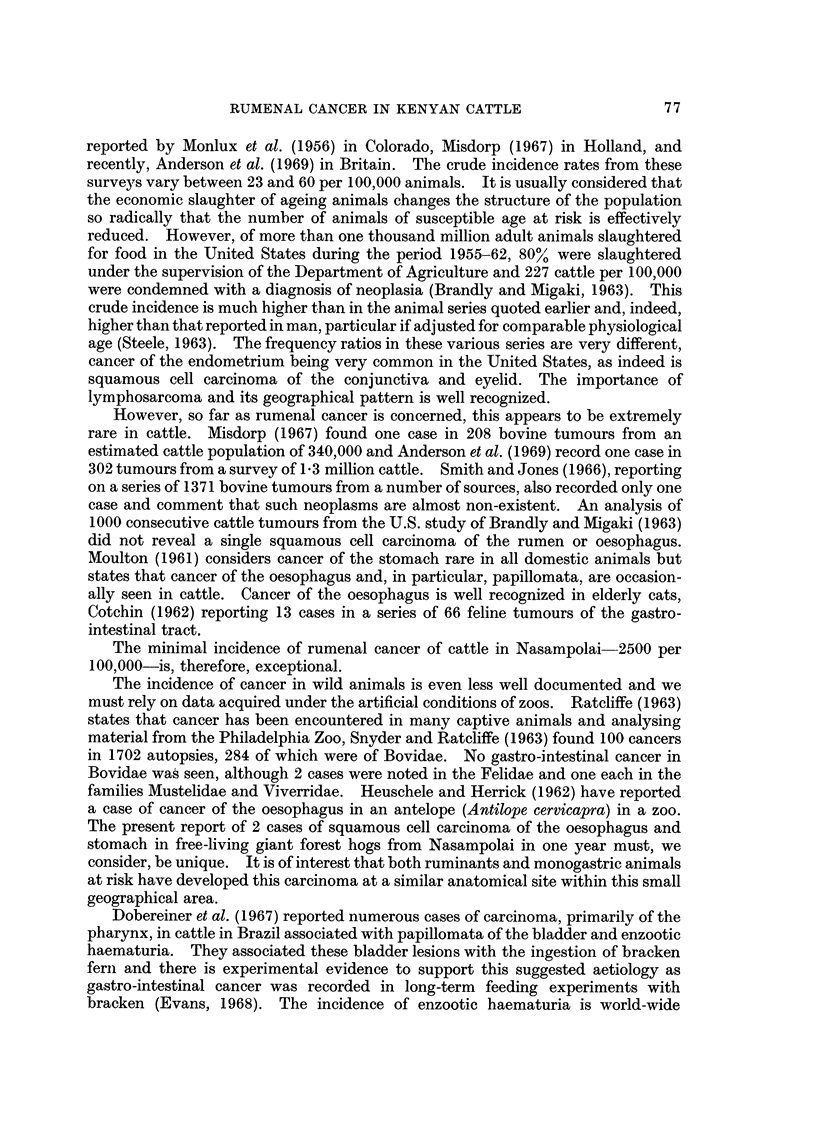

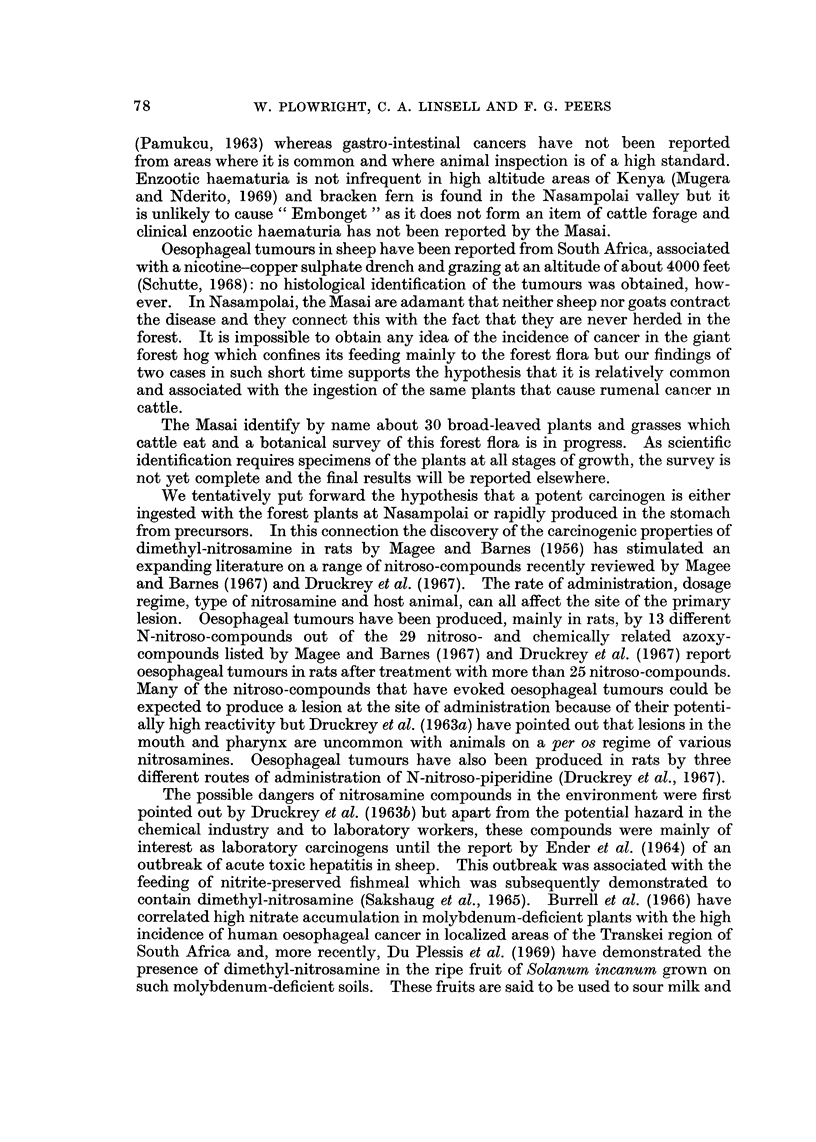

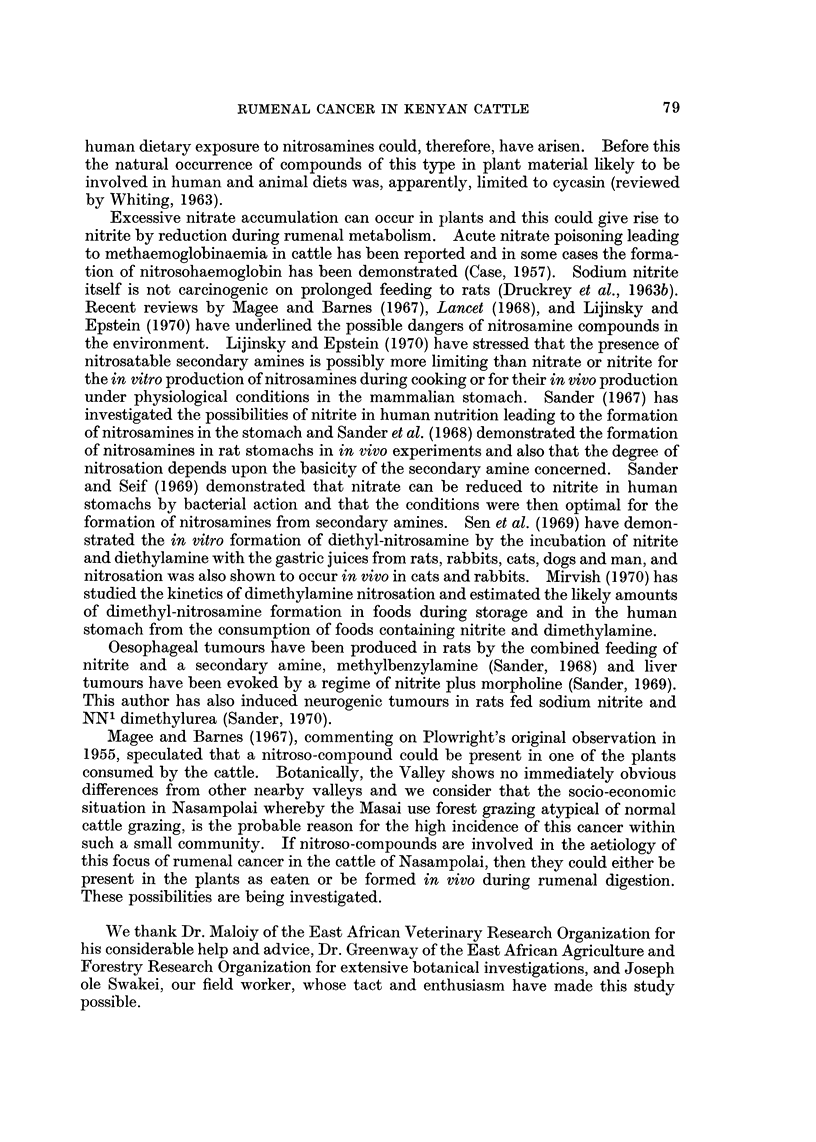

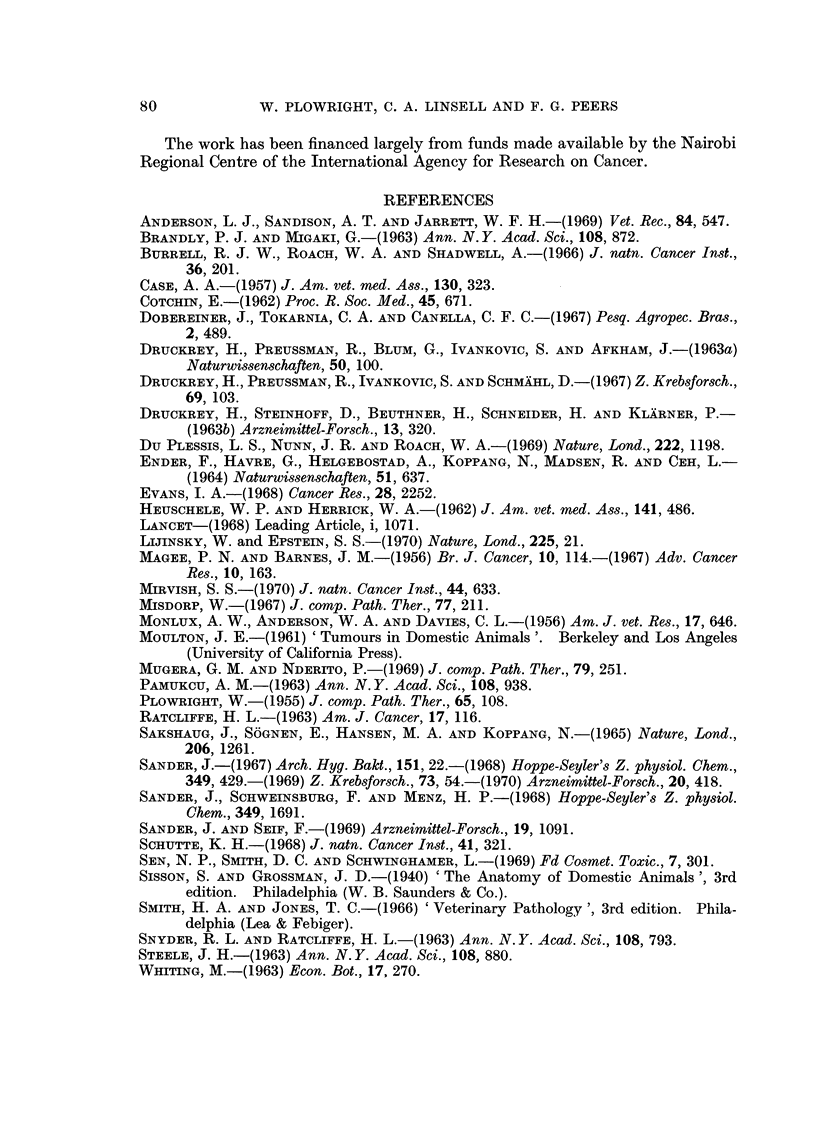


## References

[OCR_00693] ANDERSON W. A., DAVIS C. L., MONLUX A. W. (1956). A survey of tumors occurring in cattle, sheep, and swine.. Am J Vet Res.

[OCR_00648] Anderson L. J., Sandison A. T., Jarrett W. F. (1969). A British abattoir survey of tumours in cattle, sheep and pigs.. Vet Rec.

[OCR_00653] Burrell R. J., Roach W. A., Shadwell A. (1966). Esophageal cancer in the Bantu of the Transkei associated with mineral deficiency in garden plants.. J Natl Cancer Inst.

[OCR_00655] CASE A. A. (1957). Some aspects of nitrate intoxication in livestock.. J Am Vet Med Assoc.

[OCR_00656] COTCHIN E. (1952). Neoplasms in cats.. Proc R Soc Med.

[OCR_00666] Druckrey H., Preussmann R., Ivankovic S., Schmähl D. (1967). Organotrope carcinogene Wirkungen bei 65 verschiedenen N-Nitroso-Verbindungen an BD-Ratten.. Z Krebsforsch.

[OCR_00674] Du Plessis L. S., Nunn J. R., Roach W. A. (1969). Carcinogen in a Transkeian Bantu food additive.. Nature.

[OCR_00682] HEUSCHELE W. P., HERRICK W. C. (1962). Epidermoid carcinoma of the esophagus of an antelope.. J Am Vet Med Assoc.

[OCR_00688] Magee P. N., Barnes J. M. (1967). Carcinogenic nitroso compounds.. Adv Cancer Res.

[OCR_00699] PAMUKCU A. M. (1963). EPIDEMIOLOGIC STUDIES ON URINARY BLADDER TUMORS IN TURKISH CATTLE.. Ann N Y Acad Sci.

[OCR_00701] PLOWRIGHT W. (1955). Malignant neoplasia of the oesophagus and rumen of cattle in Kenya.. J Comp Pathol.

[OCR_00728] SNYDER R. L., RATCLIFFE H. L. (1963). FACTORS IN THE FREQUENCY AND TYPES OF CANCER IN MAMMALS AND BIRDS AT THE PHILADELPHIA ZOO.. Ann N Y Acad Sci.

[OCR_00705] Sakshaug J., Sögnen E., Hansen M. A., Koppang N. (1965). Dimethylnitrosamine; its hepatotoxic effect in sheep and its occurrence in toxic batches of herring meal.. Nature.

[OCR_00709] Sander J. (1968). Nitrosaminsynthese durch Bakterien.. Hoppe Seylers Z Physiol Chem.

[OCR_00713] Sander J., Schweinsberg F., Menz H. P. (1968). Untersuchungen über die Entstehung cancerogener Nitrosamine im Magen.. Hoppe Seylers Z Physiol Chem.

[OCR_00716] Sander J., Seif F. (1969). Bakterielle Reduktion von Nitrat im Magen des Menschen als Ursache einer Nitrosamin-Bildung.. Arzneimittelforschung.

